# Feasibility and Effectiveness of Electrochemical Dermal Conductance Measurement for the Screening of Diabetic Neuropathy in Primary Care. Decoding Study (Dermal Electrochemical Conductance in Diabetic Neuropathy)

**DOI:** 10.3390/jcm8050598

**Published:** 2019-05-01

**Authors:** Juan J. Cabré, Teresa Mur, Bernardo Costa, Francisco Barrio, Charo López-Moya, Ramon Sagarra, Montserrat García-Barco, Jesús Vizcaíno, Immaculada Bonaventura, Nicolau Ortiz, Gemma Flores-Mateo, Oriol Solà-Morales, the Catalan Diabetes Prevention Research Group

**Affiliations:** 1 Catalan Diabetes Prevention Research Group, IDIAP Jordi Gol., 43202 Reus, Barcelona, Spain; costaber@gmail.com (B.C.); ciscobarrio@gmail.com (F.B.); rsagarra.tarte.ics@gencat.cat (R.S.); mgarcia.tarte.ics@gencat.cat (M.G.-B.); jvizcaino.tgn.ics@gencat.cat (J.V.); gemmaflores@gmail.com (G.F.-M.); 2 Primary Care, CAP Rubí, Mutua Terrassa, 08191 Rubí, Spain; clopez@mutuaterrassa.cat; 3 Neurology Department, Hospital Mutua Terrassa, 08221 Terrassa, Spain; ibonaventura@mutuaterrassa.cat; 4 Neurology Department, Hospital Universitari Sant Joan de Reus, 43202 Reus, Spain; nortiz@grupsagessa.com; 5 HITT (Health Innovation Technology Transer), 08006 Barcelona, Spain; osola@hittbcn.com

**Keywords:** dermal electrochemical conductance, diabetes mellitus, neuropathy, primary care, screening, sudomotor reflex

## Abstract

Diabetes mellitus (DM) is the leading cause of polyneuropathy in the Western world. Diabetic neuropathy (DNP) is the most common complication of diabetes and is of great clinical significance mainly due to the pain and the possibility of ulceration in the lower limbs. Early detection of neuropathy is essential in the medical management of this complication. Early unmyelinated C-fiber dysfunction is one of the typical findings of diabetic neuropathy and the first clinical manifestation of dysfunction indicating sudomotor eccrine gland impairment. In order to assess newly developed technology for the measurement of dermal electrochemical conductance (DEC), we analyzed the feasibility and effectiveness of DEC (quantitative expression of sudomotor reflex) as a screening test of DNP in primary health care centers. The study included 197 people (with type 2 diabetes, prediabetes and normal tolerance) who underwent all the protocol tests and electromyography (EMG). On comparing DEC with EMG as the gold standard, the area under the receiver operating characteristic (ROC) curve (AUC, area under the curve) was 0.58 in the whole sample, AUC = 0.65 in the diabetes population and AUC = 0.72 in prediabetes, being irrelevant in subjects without glucose disturbances (AUC = 0.47). Conclusions: In usual clinical practice, DEC is feasible, with moderate sensitivity but high specificity. It is also easy to use and interpret and requires little training, thereby making it a good screening test in populations with diabetes and prediabetes. It may also be useful in screening general populations at risk of neuropathy.

## 1. Introduction

The prevalence of diabetes mellitus (DM) is very high in Spain, being approximately 14% according to oral glucose tolerance test (OGTT) results [1]. The management of DM requires a significant consumption of health care resources, mainly in relation to the care of vascular complications. Among the late microvascular events which may develop in patients with DM, polyneuropathy (PN) is the most common and disabling, and is the leading cause of morbidity and mortality in these patients [2]. Indeed, in Spain, the leading cause of neuropathy is DM, with its prevalence increasing with the presence of DM and other risk factors such as obesity [3]. 

PN is defined as the presence of symptoms and/or signs of peripheral nerve dysfunction in people with DM, after ruling out other possible causes [4]. The Toronto Panel Consensus on PN defined this disorder as a symmetrical, length-dependent sensorimotor PN attributed to metabolic and microvessel alterations due to chronic exposure to hyperglycemia and other risk factors. In patients with PN, thin fibers (autonomic system—sweating) and thermal and tactile sensitivity are first affected, followed by the involvement of large fibers, presenting an altered vibrating sensation which eventually alters electromyography (EMG) patterns. Therefore, dysfunction of the sweat reflex in small distal fibers is one of the earliest changes detected in these patients [5]. 

The most common clinical presentation of PN is distal symmetric polyneuropathy (DSPN), being predominantly sensory in 80% of cases [3]. Pain is the most important symptom and is described as burning or flashing, lancinanting, deep, and with frequent exacerbations during rest [4]. Pain often affects the quality of life of these patients, and it is a frequent cause of depression and/or anxiety [6]. Moreover, some patients may develop hypoesthesia, which may lead to severe foot lesions [7]. The prevalence of DSPN varies greatly according to the population, definition, and detection method. 

In the Rochester study including >64,000 patients, the prevalence of PN was between 66% and 59% for type1 DM and type 2 DM, respectively [8]. The 3rd report of the Technical Study Group of Diabetes of the World Health Organization (WHO) described a prevalence of 40% [7], and 50% in patients with >25 years of DM evolution. Pirart et al. [9] reported a prevalence ranging from 25% to 48% [7,10,11,12,13,14,15,16,17], whereas in Spain, Cabezas-Cerrato et al. published a figure of 24.1% [10].

DSPN-related factors are age, DM duration, metabolic control, male gender, acute myocardial infarction, hyperlipidemia (especially hypertriglyceridemia), smoking, and general cardiovascular risk factors [2,15,16,18]. Puig et al. [11] also included urinary albumin excretion as a risk factor of presenting DSPN. The diagnosis of DSPN is commonly made based on signs and symptoms and usually includes the use of several scores such as the Neuropathy Disability Score (NDS), the Neuropathy Symptoms Score (NSS), the Michigan Neuropathy Instrument (MNI) and the Douleur Neuropathique 4 Questions (DN4). These methods are easy to perform and are reproducible, sensitive, and adequate for use in a screening program [14].

There are many confirmatory tests, including measurements of nerve conduction velocity (EMG) and biothesiometry or skin biopsy. However, the most commonly used are the measurement of altered sensations using a vibrating tuning fork with 128 Hz and/or pressure with Semmes-Weinstein 5:07 monofilament [15]. Monofilament testing (MFT) is widely accepted and recommended by all scientific societies because of its validity, predictive risk, efficiency, and simplicity. Feng et al. [16] reported that monofilament (MFT) has a sensitivity of 57% to 93%, a specificity of 75% to 100%, a positive predictive value (PPV) of 36% to 94%, and a negative predictive value (NPV) of 84% to 100% compared with the measurement of nerve velocity by EMG. 

Although electrophysiological measures are more objective and reproducible, they are limited in that they only detect dysfunction based on the presence of thicker and faster (myelinated) fibers and show their involvement later. Consequently, EMG is a specific, albeit very insensitive, test. Recently developed noninvasive techniques are more reproducible and reliable for the detection of early dysfunction of small fibers. One of these new techniques involves the measurement of dermal electrochemical conductance (DEC) or the sudomotor dysfunction index and has been evaluated by well-designed studies which support its use as a screening test [17,18,19,20,21]. 

Ramachandran et al. [22] studied the use of DEC to detect diabetes and other disorders of glucose metabolism. In a study on the use of DEC, Casellini et al. [5] applied a PN test which showed a low sensitivity of 78% and a specificity of 92% in diabetic patients without neuropathy compared to other subjects with neuropathy and a control group. In this latter study, correlation with clinical parameters showed adequate reproducibility of the results, particularly in regard to the measurements of the feet [5]. Several other studies [23] also obtained significantly lower DEC values on comparing diabetic patients and controls. In a study of patients following a 12-month program of intense physical activity, Raisanen et al. [18] observed a greater improvement in DEC compared to weight, waist circumference, or maximum oxygen volume (VO2 max). 

Although we will not discuss the best algorithm to screen diabetic neuropathy, certain tests have shown promising results in predicting future diabetes-related complications. Some of these tests include related factors such as obesity, macroangiopathy (i.e., kidney disease) or retinopathy [24,25,26,27,28]. Therefore, taking into account the large number of methods used and the learning curve required to correctly implement these techniques, as well as the absence of consensus as to which method is the most adequate to diagnose DSPN, the aim of this study is to validate the usefulness of DEC measurement in the early diagnosis of DSPN compared with traditional techniques in the primary care setting.

## 2. Experimental Section

### 2.1. Hypothesis and Objectives

The hypothesis of our study was that the measurement of DEC is feasible, sensitive, and specific and more or equally effective to other techniques commonly used in the initial screening of diabetic neuropathy in primary care.

#### 2.1.1. Main Objective

To evaluate the feasibility, effectiveness and performance of a new technique which measures DEC (sudomotor reflex) in the screening of diabetic neuropathy in primary care.

#### 2.1.2. Specific Objectives

Determine the performance of DEC (quantitative assessment of sudomotor reflex) as a tool for the screening of diabetic neuropathy in primary care compared with the Semmes-Weinstein 5:07 MFT when an EMG is used to confirm the presence of diabetic PN in patients with prediabetes and type 2 diabetes.

Determine the performance of DEC (quantitative assessment of sudomotor reflex) as a tool for screening diabetic neuropathy in primary care compared with the Semmes-Weinstein 5:07 MFT when the NDS is used to confirm the presence of diabetic PN in patients with prediabetes and type 2 diabetes.

### 2.2. Material and Methods

The study design has been extensively described elsewhere [29]. In brief, this was a prospective study aimed at evaluating (a) the feasibility of the protocol in primary care and (b) the capacity of achieving early diagnosis of PN. A prospective and blinded comparison was made between DEC (sudomotor reflex) (Sudoscan^®^, Impeto Medical, Paris, France) and EMG; with MFT 5.07 (10 g) Semmes-Weinstein; with Rydel-Seiffer 128 Hz tuning fork (vibrating sensibility); and the NDS score, in a consecutive sample of individuals attended in the primary care setting in the Vallès Occidental and Baix Camp counties (Catalonia, Spain), and their reference hospitals.

#### 2.2.1. Design

We performed a blind, prospective study comparing DEC (sudomotor reflex) (Sudoscan™, Impeto Medical, France), EMG, the Semmes-Weinstein 5:07 MFT (10 g), the sensitivity of a vibrating tuning fork 128 Hz, NDS score and DN4 score in a consecutive series of patients treated in primary care.

#### 2.2.2. Sites

The primary care teams of Terrassa-Sud and other partners belonging to the Mútua Terrassa reference hospital and those of the primary care teams of Reus (CAP Sant Pere) and the University Hospital Sant Joan de Reus reference hospital participated in the study.

#### 2.2.3. Study Subjects

We consecutively included patients with type 2 DM over 40 years of age, with or without symptoms of neuropathy, attended in primary care. We also included the following 2 groups of patients matched by age and sex: one including patients with prediabetes (intermediate alterations of glucose metabolism defined as impaired fasting glucose (IFG)) and/or impaired glucose tolerance (IGT) determined by OGTT after 2-h 75 g oral glucose administration and another including patients without glucose alterations (normal glucose tolerance) (control group).

Three main diagnostic categories (normal, prediabetes, and diabetes) were defined using the WHO criteria based on 2-h postload glucose (<7.8 (140 mg/dL), 7.8–11.0 mmol/L (140–200 mg/dL)) and/or fasting plasma glucose (6.1–6.9 mmol/L; 110–126 mg/dL) and >11.1 mmol/L (>200 mg/dL), respectively.

The exclusion criteria were type 1 DM, upper or lower limb amputation (except phalanges), diagnosis of neuropathy not related to diabetes, neuropathy by entrapment, use of psychoactive substances, chronic alcoholism, malnutrition; treatment with beta-blockers, presence of terminal disease, or life expectancy <3 years. Pregnancy was ruled out in women (negative pregnancy test) and a history of gestational diabetes was also taken into account.

The study period was from 1 January 2017 to 31 January 2019.

#### 2.2.4. Sample Size

It was estimated that the study should include a total of 160 participants in order to evaluate the validity and performance of a screening test showing a sensitivity of 82%, a precision of 9%, and a confidence interval of 95% (95% CI: 128–192), and considering a drop-out rate of 20%. The proportion of diabetes/prediabetes/normal glucose tolerance was 2:1:1.

The contribution of patients by centers was 66% from the Mutua de Terrassa (minimum 106 cases) and 34% from Reus (minimum 54 cases), achieving a final sample size of 197 cases. 

#### 2.2.5. Variables and Data Collection

This has been described previously elsewhere [29]. In brief, a three-step procedure was carried out to obtain the study data:

##### First Visit: In Primary Care

After verifying the inclusion criteria and receiving written informed consent to participate, the medical history of the patients was obtained and a physical examination was performed using the MFT, and the NDS score was given to screen for PN. The patients also underwent DEC quantification using the Sudoscan device. A score of 8 out of 8 with the Semmes-Weinstein 5:07 MFT (10 g) was considered as sensitive [16]. An NDS score ≥6 points was considered as the presence of moderate or severe PN [30]. The determination of vibration sensitivity was performed using a 128 Hz Rydel-Seiffer tuning fork. 

##### Second Visit: In Hospital

Done at the reference hospital, where a neurologist blinded to previous test results, performed a neurographic test, including a sensory conduction study of the median, ulnar and sural nerves, and motor conduction study of the deep peroneal nerve. The variables studied included the amplitude of compound muscle action potential and distal latency of the motor nerves, and amplitude and distal latency of sensory nerves. DEC determination and the other neuropathy screening and electrophysiological tests took no longer than 1 month.

##### Third Visit: In Primary Care

The results of the previous visit were recorded and the patients were informed of the results and the diagnosis.

#### 2.2.6. Statistical Analysis

The χ^2^ test was used to analyze qualitative variables and the Student’s t test was performed for quantitative variables. Logistic regression was used to identify predictors of diabetic neuropathy. Dependent variables (response) included the presence of diabetic neuropathy diagnosed by EMG or the NDS questionnaire. The performance of DEC was compared with the MFT as screening tests of PN. To determine the validity and reliability of DEC, the sensitivity, specificity, the PPV and NPV, and the positive and negative likelihood ratios were calculated. A receiver operating characteristic (ROC) curve was used and the area under the curve was calculated. A *p* < 0.05 was considered as statistically significant. The analyses were performed with the statistical packages STATA/SE 12.0 (StataCorp, College Station, TX, USA) and R for Windows (R Foundation, Vienna, Austria).

#### 2.2.7. Ethics Approval and Consent to Participate

The research Ethics Committee board at the Jordi Gol Research Institute (Barcelona) (www.idiapjordigol.org) approved the protocol (January 2015, reference number P14/147) and each participant signed written informed consent.

#### 2.2.8. Registry

The study is registered in https://clinicaltrials.gov/ with the reference number NCT03495089.

## 3. Results

A total of 197 participants were evaluated: 100 with T2D, 50 with prediabetes and 47 controls free of glucose disturbances. 

Table 1 shows the socio-demographical and clinical characteristics of the whole sample. The three groups showed significant differences in variables usually associated with diabetes, including family history, body mass index (BMI) and waist circumference, mean systolic blood pressure and comorbidities (i.e., high blood pressure or dyslipidemia, linked to their pharmacological treatment). Differences were also observed in triglyceride values and the glomerular filtration rate. Normal Douleur Neuropathique 4 Questions (DN4) questionnaire results were more frequently observed in the control group without DM.

The prevalence of PN was 14.4% by DEC and 21% by EMG. Considering EMG as the gold standard, on evaluating the DEC in the whole sample the results were: sensitivity 21%, specificity 95%, PPV 47% and NPV 81%, with a likelihood ratio of 4.13 and an area under the receiver operating characteristic (OC) curve (AUC) of 0.58. 

The high specificity found indicates that DEC is a reliable test for the detection of individuals without PN while the low sensitivity suggests that this test is not very useful for the detection of disease among people with PN. 

On comparing DEC with other tests such as the MFT, NDS or DN4 questionnaires, the sensitivity, specificity, PPV and NPV were similar, in the whole population and in individuals without DM and in those with prediabetes (Table 2 and Table 3). 

Table 4 reports conductance (hands and feet) for the 3 different groups. All the prediabetic and normotolerant patients underwent oral glucose tolerance test since this was the method used to rule out ignored diabetes. It was therefore considered mandatory in the 97 cases included without known diabetes.

Painful neuropathy (DN4 >4 points) was observed in a total of 12 patients: 11 in the diabetes group (11%) and 1 in the prediabetics (0.02%) and none in the group with normal tolerance.

**Figure 1:**
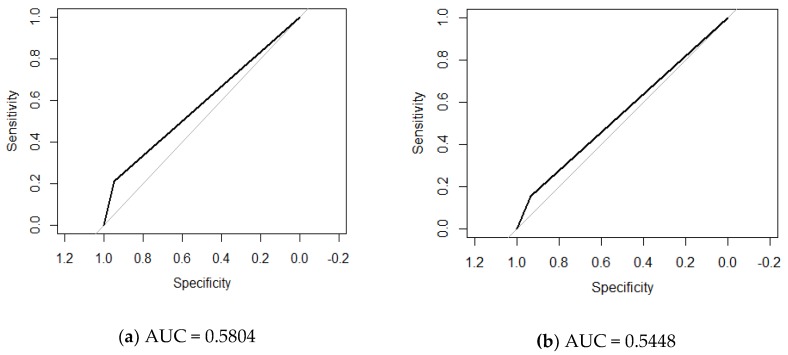
(**a**) Receiver operating characteristic (ROC) curve of dermal electrochemical conductance (DEC) versus electromyography (EMG) as the gold standard in the whole study population. (**b**) ROC curve of DEC versus monofilament (MFT) as the gold standard in the whole study population. AUC: Area under the ROC Curve.

**Figure 2:**
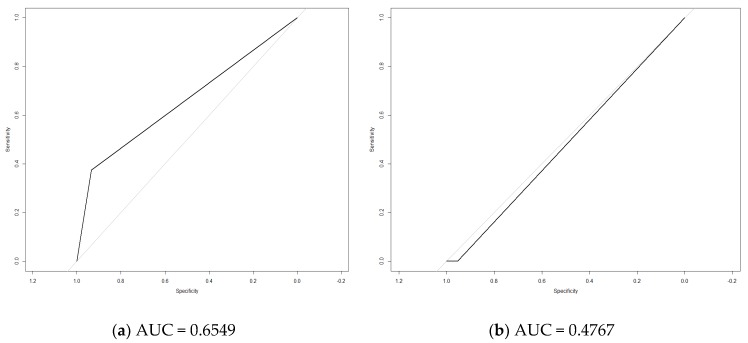
(**a**) Receiver operating characteristic (ROC) curve of dermal electrochemical conductance (DEC) versus electromyography (EMG) as the gold standard in the population with diabetes mellitus. (**b**) ROC curve of DEC versus EMG as the gold standard in the population without glucose disturbances. AUC: Area under the ROC curve.

**Figure 3:**
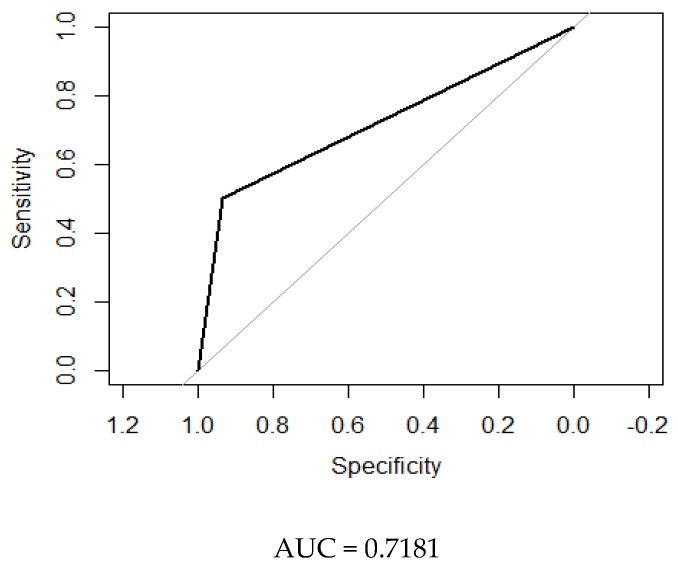
Receiver operating characteristic (ROC) curve of DEC versus electromyography (EMG) as the gold standard in the population with prediabetes.

Figure 1, Figure 2 and Figure 3 show the ROC curves comparing DEC versus the other tests evaluated. 

In the whole sample, the ROC curve comparing DEC with MTF (AUC = 0.65) showed greater effectiveness than that comparing DEC vs. EMG (AUC = 0.54). On the other hand, in the population with type 2 diabetes, the comparison of DEC with EMG showed the greatest significance (AUC = 0.66). Although the results are not very high, DEC may be a good screening test for populations with PN compared to the gold standard (EMG). 

It was of note that in the screening of prediabetic subjects the AUC was 0.72, which is reasonable for a screening test. 

## 4. Discussion

As mentioned in the Introduction, state-of-the-art methods demonstrate a high variability in PN diagnostic methods, being directly related to the variability in prevalence rates. For example, with the use of EMG, Puig and Portillo obtained prevalences of 75 and 86%, respectively [11,25]. However, most authors use clinical methods based on exploratory symptoms and signs, obtaining diverse prevalences ranging from 13% up to 81% [10,12,14,26,27,28]. It is clear that the tests used to determine the diagnosis of PN and the type of patients included have an impact on the prevalence, as does the cut-off to consider pathology. Gordon Smith [17] found poor DEC results in feet with PN, reporting a sensitivity higher than in our study (77%) but with a lower specificity (67%) and with a higher cut-off (70 µS vs. 60 µS, respectively). Casellini [5] compared three groups (diabetic individuals with and without PN, and healthy volunteers) and also concluded that DEC is less effective in diabetic subjects with PN, showing similar results to our study (sensitivity 78%, specificity 92%). In a study of 75 individuals, but including 45 patients with type 1 diabetes, Selvarajah [23] also described similar results with DEC (sensitivity 87%, specificity 76% and AUC 0.85). Nonetheless, unlike our study, these three studies included subjects with known confirmed (or discarded) PN. The aim of the present study was to validate DEC as a method to diagnose PN, and the presence of PN or causes other than diabetes were an exclusion criterion. Eranki [20] used DEC for the detection of cardiovascular complications in 308 patients with diabetes, reporting complications in 120 cases, 79% due to neuropathy. They reported a sensitivity of 83%, a specificity of 55% and an AUC = 0.73 for PN. The results of the study by Erank et al. differed greatly from ours, which showed a very low sensitivity but a very high specificity due to the low number of cases. 

One important difference of our study is that we also evaluated a group with prediabetes. With the addition of this group, the definitive results differ from the preliminary results, with the NDS questionnaire showing the best AUC compared to MFT before the addition of prediabetes. There are few studies in this regard. However, some have suggested the utility of DEC for detecting diabetes in subjects with glucose intolerance, being even better than basal glycemia. With the use of DEC in 212 subjects, Ramachandran [22] diagnosed 24 type 2 diabetes, 30 cases with glucose intolerance and 57 subjects with normal glucose tolerance but with metabolic syndrome.

In a study evaluating 47 patients with type 2 diabetes and 16 controls, Kneger et al. [31] reported very similar results to those of the present study. DEC was effective in detecting subjects with PN and correlated well with clinical signs and symptoms of neuropathy. 

Bins-Hall et al. [32] studied a one-step approach for the diagnosis of PN in 236 patients with diabetes undergoing funduscopy. These authors evaluated a battery of tests including: the Toronto scale, MFT, and two devices: DPN-Check™ and Sudoscan™. The results showed high performance (with the Toronto scale as the gold standard (30.9%)) with DPN-check (sensitivity 84.3%, specificity 68.3%) and Sudoscan (sensitivity 77.4%, specificity 68.3%) compared to MFT (underestimation, only 14.4% detection). 

In a Chinese cohort, Sudoscan™ was a useful method to detect PN in a screening of 394 asymptomatic persons with diabetes [33].

In a systematic review and meta-analysis on MFT, Wang et al. [34] reported that the sensitivity of this test was very low. 

It is of note that in present study we prioritized the NDS and DN4 scores; the first provides clinical data (questionnaire) and exploration data (vibration, temperature, reflexes) while the second is an abbreviation of the first, and provides a greater amount of clinical data. 

### Limitations and Strengths of the Study

The main limitation of the present study is accurate diagnosis of diabetic neuropathy since some studies have shown that some cases of diabetic neuropathy present no alterations in the EMG. 

Indeed, both EMG and MFT examine large nerve fibers. However, since they are the usual tests available, they represent a limitation of the study. Therefore, several considerations should be taken into account. First, the EMG test is more specific, albeit not very sensitive, showing positive results in advanced stages of PN. The fingerboard and the NDS questionnaire are commonly used for the diagnosis of diabetic neuropathy, probably because the NDS is carried out before EMG and is actually often used to avoid the need for EMG.

Another inherent limitation of this study may be the low number of participants, although this number fulfilled that obtained in the calculation of the sample size. 

Therefore, both the EMG and the NDS score, which mainly assess the dysfunction of myelinated fibers, provide a good profile for diagnostic confirmation. On the contrary, both the DEC and the MFT are able to diagnose and stage diabetic neuropathy earlier than the previous 2 tests by the detection of unmyelinated fiber dysfunction. Nonetheless, another limitation is that MFT is a good test for the prediction of foot ulcers but is certainly insensitive for the detection of early neuropathy. Therefore, for purposes of simplification and taking into account the possible limitations, we compared the effectiveness of the measurement of DEC and the use of MFT as diagnostic tools in primary care according to whether the true diagnosis is achieved by the EMG or the score of the NDS questionnaire.

Although other tests are available to detect early neuropathy, such as the Utah Early Neuropathy Scale (UENS) and the Norfolk Quality of Life Questionnaire-Diabetic Neuropathy (QOL-DN) scale, these questionnaires are not practical in the clinical scenario. However, the latter test is a good tool to detect neuropathy unawareness in patients with diabetes [35]. Indeed, in the study by Veresiu et al., the Norfolk QOL-DN scale was used in 25,000 patients with diabetes and it was found that neither 6600 patients nor their physicians were aware of the presence of neuropathy. 

In regard to technical aspects, the use of DEC in clinical examinations has a few limitations as described in previous studies (the use of drugs such as tricyclic antidepressants, non-cardioselective beta-blockers, or extensive dermatitis on the palms of the hands or soles of the feet). However, we do not believe that these aspects apply to the present study since they were exclusion criteria and were not affected by age, sex or previous physical exercise or changes in temperature or acute alterations in blood pressure. According to these studies, the results of DEC do not depend on the rate of perspiration [36], having a high correlation (correlation coefficient 0.814; coefficient of variation 1.15%) [5].

One of the strengths of this study was that DEC and physical examinations were performed by only a few investigators in order to avoid biases in sample selection or erroneous categorization of the diagnosis of PN. This is one of the first studies to compare DEC measurement with the gold standard (EMG). Other studies, even those by the manufacturer, are based on other devices or clinical questionnaires, thereby making our study innovative. 

Considering the limitation of resources in primary care and the health care system as a whole, we are currently performing a cost-effectiveness analysis of the use of DEC in the primary care setting. 

## 5. Conclusions

In conclusion, in the usual clinical practice, DEC is feasible, with a moderate sensitivity but a high specificity. It is also easy to use and interpret and requires little training, thereby making it a good screening test in populations with diabetes and prediabetes. It may also be useful in screening general populations at risk of neuropathy. 

Nonetheless, the results of cost-effectiveness analyses must first be analyzed before recommending the implementation of DEC in primary care in Catalonia. 

## Figures and Tables

**Table 1:** Sociodemographic and clinical data of the study participants (type 2 diabetes (T2D), prediabetes and normal glucose tolerance).

Variable	Participants with T2D (*n* = 100)	Participants with Prediabetes (*n* = 50)	Participants with Normal Glucose Tolerance (*n* = 47)	*p*
Age (mean ± SD)	64.65 ± 7.5	62.7 ± 6.8	63.47 ± 7.4	0.30
Gender (% men)	50	38	38.3	0.33
Origin country (% Spain)	93	100	93.6	0.70
Family history of diabetes (%)	83 (83)	26 (50.2)	33 (70.2)	<0.001
Current smoker; *n* (%)	8 (8)	2 (4)	3 (6.4)	0.99
Alcohol consumption; *n* (%)	59 (59)	34 (68)	30 (63.8)	0.70
T2D evolution (mean ± SD, year)	10.6 ± 7.52	-	-	-
T2D complications (%)				
Cardiopathy	4	1	0	-
retinopathy	6	0	0	-
peripheral vascular disease	7	0	0	-
stroke	3	0	1	-
nephropathy	6	0	0	-
NIAD treatment (*n*)	152	-	-	-
Insulin treatment (*n*)	29	-	-	-
Antihypertensive treatment; *n* (%)	74 (74)	28 (56)	25 (53.1)	0.02
Cholesterol-lowering agents; *n* (%)	62 (62)	18 (36)	15 (31.9)	0.001
Antiaggregant treatment; *n* (%)	21 (21)	8 (16)	6 (12.7)	0.33
Height (mean ± SD, cm)	162.4 ± 9.7	162.7 ± 8.8	160.8 ± 7.5	0.54
Weight (mean ±SD, kg)	79.6 ± 13.6	85.3 ± 18.3	74.9 ± 14.9	0.004
BMI (mean ±SD, kg/m^2^)	30.3 ± 4.5	33.28 ± 10.8	28.6 ± 4.6	0.003
Waist circumference (mean ± SD, cm)	105.0 ± 11.2	108,0 ± 15.3	109.5 ± 11.4	0.016
SBP (mean ± SD, mmHg)	136.5 ± 14.1	132.3 ± 13.6	125.8 ± 15.2	<0.001
DBP (mean ± SD, mmHg)	78.2 ± 10.4	79.8 ± 7.9	75.3 ± 9.5	0.06
HbA1c (mean ± SD, %)	7.2 ± 1.2	5.9 ± 0,3	5.3 ± 0.4	0.02
Glomerular Filtration Rate (mean ± SD, mL/min/1.73 m^2^)	67.3 ± 14.4	87.0 ± 7.3	72.9 ± 19.3	<0.001
Total cholesterol (mean ± SD, mg/mL)	191.9 ± 39.4	196.8 ± 33.9	190.9 ± 30.9	0.67
HDL-chol (mean ± SD, mg/dL)	48.7 ± 11.0	58.9 ± 14.5	54.9 ± 12.2	<0.001
LDL-chol (mean ± SD, mg/dL)	111.7 ± 33.3	114.4 ± 32.0	116.5 ± 28.0	0.69
Triglycerides (mean ± SD, mg/dL)	159.6 ± 90.0	120.7 ± 49.2	117.0 ± 44.7	<0.001
DN4 questionnaire (% score = 0)	61	70	74.5	<0.001

SD: standard deviation; DM: diabetes mellitus; NIAD: non-insulin antidiabetic drugs; BMI: body mass index; SBP: systolic blood pressure; DBP; diastolic blood pressure; HbA1c: glycohemoglobin A1c; HDL-chol: high density lipoprotein cholesterol; LDL-chol: low density lipoprotein cholesterol.

**Table 2:** Sensitivity (Sen), specificity (Spe), positive predictive value (PPV), negative predictive value (NPV) of dermal electrochemical conductance (DEC) compared to different tests as the gold standard.

	Gold Standard			
	EMG	MFT	NDS	DN4
**DEC**	Sen = 21.2%	Sen = 15.3%	Sen = 33.3%	Sen = 21.4%
	Spe = 94.8%	Spe = 93.5%	Spe = 94%	Spe = 93.4%
	PPV = 46.6%	PPV =26.6%	PPV = 26.6%	PPV = 20%
	NPV = 81.3%	NPV = 87.9%	NPV = 95.6%	NPV = 93.4%
	LR+ = 4.13	LR+ = 2.35	LR+ = 5.55	LR+ = 3.24
	LR− = 0.83	LR− = 0.90	LR− = 0.71	LR− = 0.77

DEC: dermal electrochemical conductance; EMG: electromyography; MFT: monofilament; NDS: Neuropathy Disability Score; DN4: Douleur Neuropathique-4 Questions; Sen: sensitivity; Spe: specificity; PPV: positive predictive value; NPV: negative predictive value. LR+: positive likelihood ratio; LR−: negative likelihood ratio.

**Table 3:** Comparison between dermal electrochemical conductance (DEC) results and other tests such as the gold standard based on the glycemic state of the participants.

		Gold Standard											
		MFT			EMG			NDS			DN4		
		DM	No-DM	Pre-DM	DM	No-DM	Pre-DM	DM	No-DM	Pre-DM	DM	No-DM	Pre-DM
DEC	Sen	25%	0%	14%	20%	0%	5%	37%	0%	1%	30%	0	0
	Spe	93%	95%	93%	96%	95%	93%	93%	95%	93%	93%	95.5%	91%
	PPV	33%	0%	25%	67%	0%	25%	33%	0%	25%	33%	0	0
	NPV	90%	84%	87%	74%	98%	96%	94.5%	93%	1%	92%	95.5%	93%
	LR+	3.57	0	2	5	0	0.71	5.28	0	0.14	4.28	0	0
	LR−	0.80	20	0.92	0.83	20	1.02	0.67	20	1.06	0.75	22.2	11.1

DEC: dermal electrochemical conductance; EMG: electromyography; MFT: monofilament; NDS: Neuropathy Disability Score; DN4: Douleur Neuropathique-4 Questions; Sen: sensitivity; Spe: specificity; PPV: positive predictive value; NPV: negative predictive value; DM: type 2 diabetes; No-DM: non-diabetic; pre-DM: prediabetic. LR+: positive likelihood ratio; LR−: negative likelihood ratio.

**Table 4:** Comparison between DEC results in the three groups of participants.

		Conductance (µS)		
		Normal Tolerance	Pre-DM	DM
**DEC**	H-DEC (hands)	70.3 ± 12.7	63.9 ± 19.2	51.8 ± 17.2
	F-DEC (feet)	76.8 ± 15.8	73.3 ± 22.5	71.3 ± 18.0
